# Neural bystander damage by infiltrating virus-infected T cells and the cytotoxic T lymphocytes in HTLV-I-associated neurological disease

**DOI:** 10.1186/1742-4690-8-S1-A120

**Published:** 2011-06-06

**Authors:** Eiji Matsuura, Ryuji Kubota, Yuetsu Tanaka, Hiroshi Takashima, Shuji Izumo

**Affiliations:** 1 Department of Neurology and Geriatrics, Kagoshima University Graduate School, Kagoshima, Japan; 2 Center for Chronic Viral Disease, Kagoshima University Graduate School, Kagoshima, Japan; 3 Department of Immunology, University of the Ryukyus, Okinawa, Japan

## 

We hypothesized that the cytotoxic T lymphocytes(CTLs) play a pivotal role in the pathogenesis of human T lymphotropic virus type I (HTLV-I)-associated myelopathy/tropical spasticparaparesis(HAM/TSP).

One of the most striking features of the cellular immune response in the patients with HTLV-I-associated myelopathy/tropical spastic paraparesis (HAM/TSP) is the highly increased numbers of HTLV-I-specific cytotoxic T lymphocytes (CTLs) in the circulation of the blood and the cerebrospinal fluid, nevertheless HTLV-I proviral load in the PBMC remains high in the patients.

To determine the CTL’s association with the pathogenesis of HAM/TSP in the CNS, we developed novel methods of in-situ detection for HTLV-I-specific CTL using HLA/antigen peptide tetramer and for the cells expressing HTLV-I viral protein. We visualized the HTLV-I specific CTLs and HTLV-I-infected cells in autopsied spinal cords of the patients with HAM/TSP.

We demonstrated that HTLV-I-specific CTLs expressing cytotoxic molecules accumulated in the spinal cords from three patients with HAM/TSP and that HTLV-I exclusively infected CD4 positive T lymphocytes but neither resident cells nor macrophages. The phenotype of apoptotic cells was HTLV-I infected CD4+ T lymphocytes or HTLV-I non-infected oligodendrocytes.

The findings suggest a unique pathogenesis for the neuroinflammatory disease that an inflammation of the central nervous system is attributed to the interaction between HTLV-I-infected CD4+ T cells and HTLV-I-specific CD8+ CTLs from the periphery.

